# Genomic Association Study for Cognitive Impairment in Parkinson's Disease

**DOI:** 10.3389/fneur.2020.579268

**Published:** 2021-02-04

**Authors:** Kye Won Park, Sungyang Jo, Mi Sun Kim, Sang Ryong Jeon, Ho-Sung Ryu, Jinhee Kim, Young-Min Park, Seong-Beom Koh, Jae-Hong Lee, Sun Ju Chung

**Affiliations:** ^1^Department of Neurology, Asan Medical Center, University of Ulsan College of Medicine, Seoul, South Korea; ^2^Department of Neurosurgery, Asan Medical Center, University of Ulsan College of Medicine, Seoul, South Korea; ^3^Department of Neurology, Kyungpook National University Hospital, Daegu, South Korea; ^4^Department of Neurology, Korea University Guro Hospital, Seoul, South Korea; ^5^Department of Neurology, Dobong Hospital, Seoul, South Korea

**Keywords:** Parkinson's disease, cognitive impairment, genome-wide association studies, dementia, ryanodine receptor

## Abstract

**Background:** Cognitive impairment is very common in Parkinson's disease (PD) and constitutes the most debilitating complication of this disease. However, to date, few studies have investigated a genome-wide association in the development of cognitive impairment of PD. We aimed to identify the genetic loci associated with cognitive impairment in patients with sporadic PD by ethnicity-specific genotyping.

**Materials and methods:** We recruited 1,070 patients with PD and performed a genome-wide association study using the Korean Chip, a microarray chip containing 827,400 single-nucleotide polymorphisms (SNPs) optimized for the Korean population. Multiple logistic regression models adjusting for age, sex, years of education, and disease duration were used to compare between patients with and without cognitive impairment, which was defined using the Mini-Mental Status Examination (MMSE) score (MMSE score ≥ 26 vs. < 26) or the Montreal Cognitive Assessment (MoCA) score (MoCA score ≥24 vs. < 24).

**Results:** *RYR2* SNP rs10495397 was most significantly associated with cognitive impairment based on the MMSE scores (OR = 3.21; 95% CI = 1.96–5.25, *P* = 3.36 × 10^−6^) and *CASC17* showed the strongest association with cognitive impairment based on the MoCA scores. However, none of the SNPs were statistically significant after Bonferroni correction.

**Conclusion:** *RYR2* may play a role in cognitive impairment in PD by the pathogenic mechanism of neuroinflammation. However, more studies are needed to replicate and validate the results of our functional study.

## Introduction

Parkinson's disease (PD) is the most common neurodegenerative movement disorder and is associated with non-motor symptoms. Cognitive impairment (CI) is the most debilitating non-motor symptom of PD. In combination with both motor symptoms and dementia, the disease imposes a significant social and economic burden on both the caregivers and society (1). Thus, there have been demands to identify the cause and risk factors of CI in individuals with PD (2, 3).

During the past few decades, genome-wide association studies (GWAS) have provided a greater understanding on the genetic background of PD, reporting about 90 SNPs associated with the disease (4, 5). However, most previous GWAS on PD primarily focused on susceptibility to the disease. Because PD is a highly heterogeneous disease, new GWAS are needed to identify genes and genetic variants associated with various clinical characteristics of PD. In terms of CI, some GWAS investigated the susceptibility of PD dementia (PDD) or dementia with Lewy bodies (DLB). These studies were primarily case–control studies that compared genetic profiles of patients with PDD or DLB to those of healthy controls (6). However, there have been limited GWAS that aimed to identify genes associated with the development of CI within the heterogeneous PD population. Furthermore, the results of previous GWAS were largely based on the western population, whereas few studies have assessed the Asian population.

In this study, we aimed to investigate the genetic loci associated with CI in Korean patients with sporadic PD.

## Methods

### Participants and Cognitive Assessment

We enrolled 1,070 patients with PD at the Asan Medical Center, Seoul, South Korea, from January 2011 to April 2016. All study subjects were born and resided in South Korea. Patients were diagnosed with PD based on the United Kingdom Parkinson's Disease Brain Bank Criteria by a movement disorder specialist. All patients underwent peripheral blood sampling for DNA extraction, and their electronic medical records were retrospectively reviewed. Baseline data included age, sex, total years of education, and disease duration. The Mini-Mental Status Examination (MMSE) and Montreal Cognitive Assessment (MoCA) scores were obtained from patients' electronic medical records. The cutoff value for CI was set to MMSE score <26 and MoCA score <24 (7, 8). The study was approved by the Institutional Review Board of Asan Medical Center. All patients provided informed consent for the study.

### Genotyping and Quality Control

All samples were genotyped using the Korean Chip (K-CHIP) designed by the Korean Chip Consortium, Center for Genome Science, Korea National Institute of Health, Korea (4845–301, 3000–3031). The K-CHIP is a microarray chip containing 827,400 single-nucleotide polymorphisms (SNPs) optimized for the Korean population. Its genomic coverage is more than 95% for SNPs of minor allele frequency (MAF) > 5% and 73% for those of MAF 1–5% in the Asian population (9).

Low-quality samples with low call rate, excessive heterozygosity, outliers in multidimensional scaling, excessive singletons, cryptic first-degree relatives, and sample with gender discrepancies were excluded using PLINK software (version 1.90, NIH-NIDDK Laboratory of Biological Modeling, Bethesda, MD, USA) and SNPolisher package of R software (version 3.1.2, Free Software Foundation, Inc., Boston, MA, USA). Cluster quality control (QC) was manually performed for every SNP with *P* < 0.001, with linkage disequilibrium within 150 kilobase through visual inspection. For marker QC, SNPs with genotyping call rate ≥95% for both groups with and without CI were included, while those with MAF <1% for both groups, and those that did not fulfill Hardy–Weinberg Equilibrium (HWE) (*P* < 10^−6^) were excluded.

### Statistical Analysis

Demographic and clinical characteristics were compared using Student's *t*-test for continuous variables and chi-square test for dichotomous variables.

We performed genetic association analysis using multiple logistic additive models to compare patients with PD with and without CI using both the MMSE score (MMSE score <26 vs. ≥26) and the MoCA score (MoCA score <24 vs. ≥24). Level of education can affect the cognitive performance of an individual, and disease duration at the time of cognitive testing can bias the results because the risk of dementia rises as the disease progresses. Therefore, we adjusted years of education and disease duration at the time of MMSE or MoCA as covariates, as well as age and sex. *P*-values from the primary analyses were assessed for significance using Bonferroni correction for multiple comparisons. Data analysis was performed using PLINK software (version 1.90, NIH-NIDDK Laboratory of Biological Modeling, Bethesda, MD, USA).

## Results

Among our sample of 1,070 patients with PD, 20 were excluded during the sample QC step, and finally, our study population included 1,050 patients. Analyses for the MMSE and MoCA scores were available for 1,029 and 494 patients, respectively.

### Association Analysis Between Genomic Variants and the MMSE Scores

Among 1,029 patients assessed using the MMSE scores, 347 with PD had score <26 and 682 had score ≥26. Regarding clinical characteristics, female sex, later age at onset of PD, lower education level, and prolonged disease duration were associated with lower MMSE scores. The APOE ε4 carrier status was similar between the two groups (Table 1).

**Table 1:** Baseline characteristics of participants.

**Characteristics**	**MMSE score <26 (*n* = 347)**	**MMSE score ≥26 (*n* = 682)**	***P-value***	**MoCA score <24 (*n* = 222)**	**MoCA score ≥24 (*n* = 272)**	***P-value***
MMSE score	22.2 ± 3.3 [10–25]	27.9 ± 1.3 [26–30]	<0.001	–	–	–
MoCA score	–	–	–	17.9 ± 4.7 [3–23]	26.5 ± 1.8 [24–30]	<0.001
Female, *n* (%)	216 (62)	330 (48)	<0.001	129 (58)	141 (52)	0.164
Age at onset, years	61.7 ± 9.5 [31–76]	57.2 ± 10.2 [30–87]	<0.001	61.3 ± 9.0 [28–87]	55.3 ± 10.3 [29–84]	<0.001
Years of education	6.0 ± 5.3 [0–23]	10.0 ± 5.9 [0–20]	<0.001	6.6 ± 5.8 [0–20]	10.5 ± 5.8 [0–20]	<0.001
Disease duration, years	6.0 ± 4.5 [1–27]	4.8 ±4.0 [1–26]	<0.001	8.7 ± 5.0 [1–28]	7.1 ±4.5 [1–26]	<0.001
ApoE4 carrier, *n* (%)	66 (19)	126 (18)	0.832	41 (18)	46 (17)	0.651

*Data are presented as mean ± standard deviation [range] or number of patients (%)*.

*MMSE, Mini-Mental Status Examination; MoCA, Montreal Cognitive Assessment; ApoE4, Apolipoprotein E4*.

**Figure 1:**
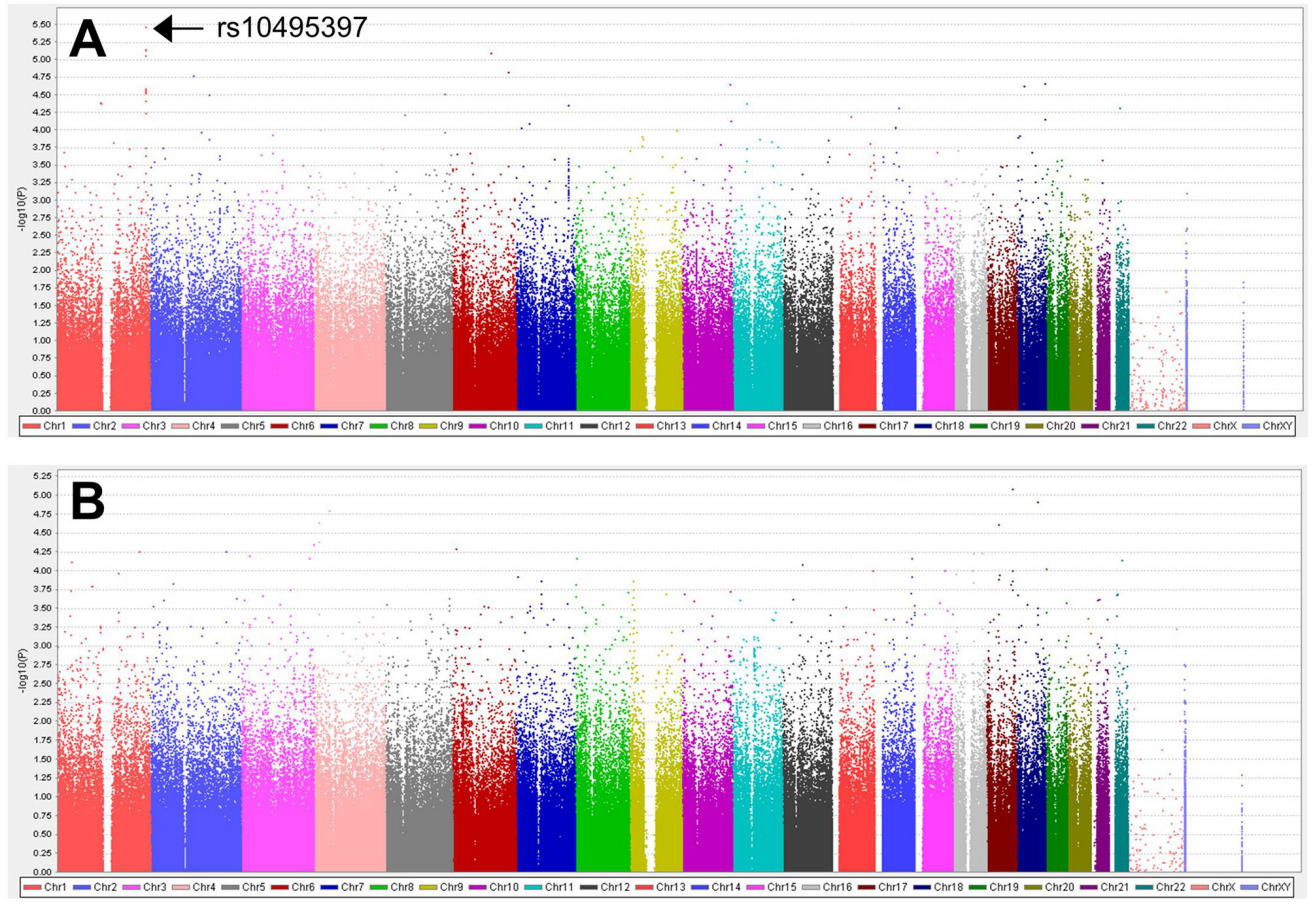
Manhattan plots for **(A)** the analysis of 563,715 SNPs between patients with MMSE score <26 vs. ≥26 and **(B)** the analysis of 523,758 SNPs between patients with MoCA score <24 vs. ≥24.

**Figure 2:**
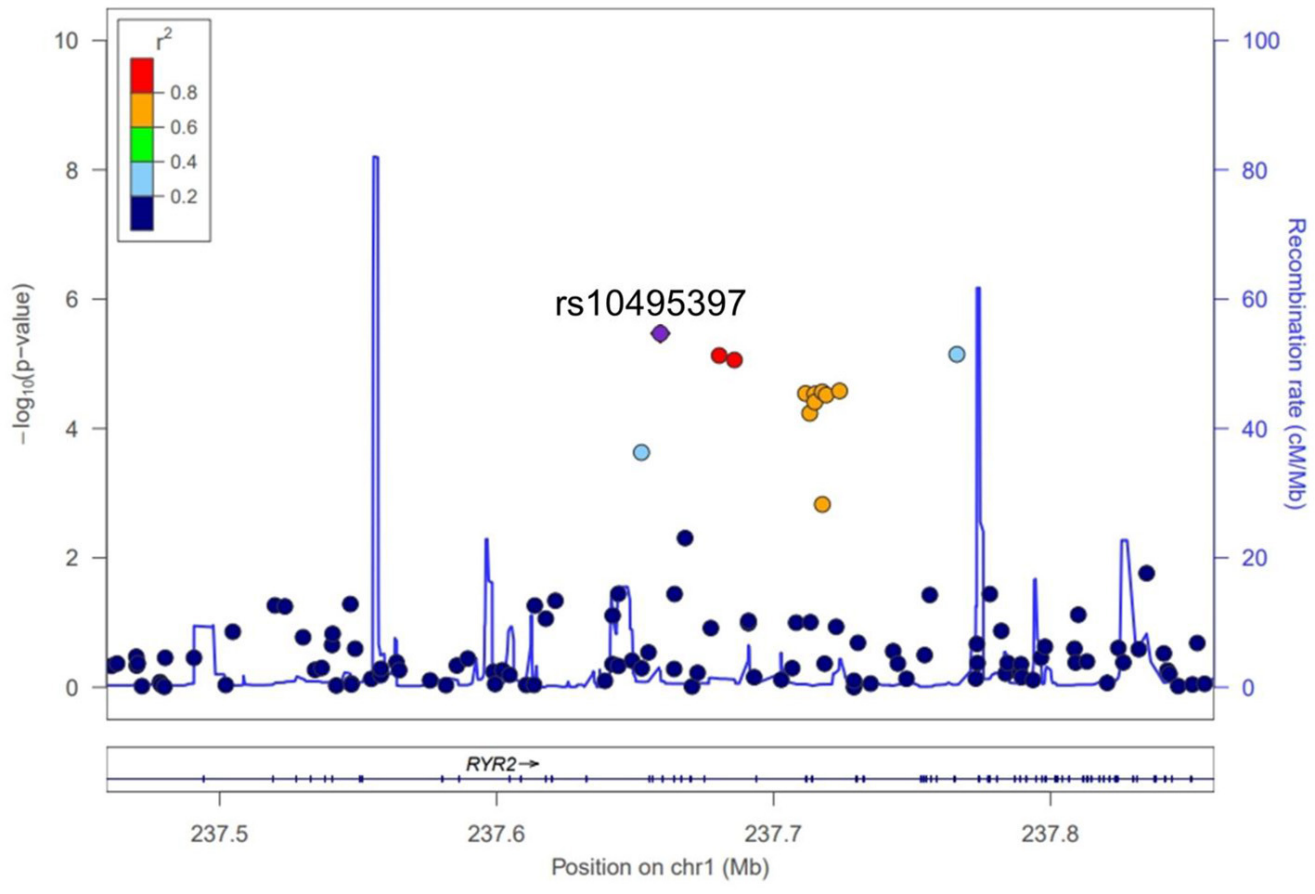
Regional association plot of the genetic variants of *RYR2*.

A total of 563,715 SNPs passed marker QC. Multiple logistic regression and additive models with covariates age, sex, education years, and disease duration were used to compare between patients with PD with MMSE score <26 and those with MMSE score ≥26. Three SNPs within the *RYR2* (rs10495397, rs78955454, and rs12745344; Figure 1A) gene had the lowest *P*-values with *P* < 10^−5^. Altogether, 10 SNPs within the *RYR2* gene were ranked among the top 20 SNPs with lowest *P-*values. In linkage analysis, these *RYR2* SNPs were in moderate to high linkage disequilibrium with the SNP with the lowest *P*-value, rs10495397 (Figure 2). However, none of the SNPs were statistically significant after Bonferroni correction (threshold of *P* 0.05/563,715 = 8.87 × 10^−8^) (Figure 1). The profile of the SNPs and genes with the strongest association and their physiologic roles in CI in PD are presented in Supplementary Table 1.

### Association Analysis Between Genomic Variants and MoCA Scores

Among 594 patients with PD who were assessed using the MoCA scores, 222 had score <24 and 272 had score ≥24. Later age at onset of PD, lower education level, and prolonged disease duration were associated with lower MoCA scores (Table 1).

A total of 523,758 SNPs passed marker QC. Multiple logistic regression and additive models with covariates age, sex, education years, and disease duration were used to compare between patients with PD with MoCA <24 and those with MoCA ≥24. *CASC17* SNP rs2430514 had the lowest *P* value (OR =2.1, 95% CI = 1.51-2.91, *P* = 8.17 × 10^−6^). However, none of the SNPs were statistically significant after Bonferroni correction (Figure 1B). The profile of the SNPs and genes with the strongest association and their physiologic roles in CI in PD are presented in Supplementary Table 2.

## Discussion

In this study, we investigated genomic variants associated with CI in patients with PD. The SNP rs10495397, a variant of the *RYR2* gene, was the most significantly associated with CI based on the MMSE scores in patients with PD with biological plausibility. The SNP rs2430514, a variant of the *CASC17* gene, showed the most significant association with CI based on the MoCA scores in patients with PD. Although these SNPs and other variants were not statistically significant after strict Bonferroni correction, the role of *RYR2* and other genes associated with CI in PD identified in this GWAS should be investigated further.

In addition to rs10495397, 9 more SNPs from the introns of *RYR2* were ranked among the 20 genes most associated with a lower MMSE score in patients with PD and were in moderate to high linkage disequilibrium with each other. The high ranking of multiple SNPs from a single gene and its high level of linkage disequilibrium are suggestive that the gene plays a significant role in cognitive function in patients with PD. *RYR* is a family of genes that encode the ryanodine receptor (RyR), which is primarily expressed in heart muscle but also plays a crucial role in neuroinflammation (10). RyR activity is altered in age-associated memory impairment, and the pharmacological blockade of RyR by dantrolene improved spatial memory and reduced age-associated increase in microglial activation in both *in vitro* and rat models (10–12). In addition, RyR antagonists are associated with improvement of abnormal calcium regulation of the neurons in Alzheimer's disease (AD) and are proposed as a therapeutic drug for AD (12). These findings suggest the important role of *RYR2* in CI in patients with PD in the context of neuroinflammation in the brain. Little is known about the physiologic role of Cancer Susceptibility Candidate 17 (*CASC17*) gene, where the SNP rs2430514 is located. However, interestingly, several previous GWAS have identified SNPs in *CASC17* associated with the risk of psychosis and impulsive behavior, which are characteristic features of dementia in PD (13, 14).

The other SNPs with the highest association with CI that were identified in this study are also linked to other physiologic functions that may play a role in CI in PD, including cellular membrane integrity, postsynaptic density regulation, regulation, neurotransmission, and a role in the axon-guidance pathway, as shown in the Supplementary Tables 1, 2.

CI in this study was defined using the MMSE and MoCA scores. This approach has some strengths and weaknesses. MMSE is the most popular and simple tool to assess cognition in a general neurology clinic. The MoCA is the most efficient tool to screen for CI in the PD population. Currently, it is recommended that the clinical diagnosis of dementia in PD be based on the Movement Disorder Society (MDS) criteria (15). However, a comprehensive neuropsychological assessment, including multiple tests in each major cognitive domain, is required to fulfill the MDS criteria, which is time- and cost-consuming for studies requiring a large number of participants, such as GWAS. Therefore, previous large-scale genetic studies in PD also favored simple tools to assess CI, including MMSE, MoCA, or *The Diagnostic and Statistical Manual of Mental Disorders*-IV criteria (16). It is also notable that there was no overlap between the set of the most statistically significant SNPs identified by MMSE and MoCA. This may reflect the different screening ability of the two tools to detect the CI itself or the impairment in specific cognitive domain. Compared with MoCA, MMSE has a weakness in assessing frontal/executive function, which is an important feature of CI in PD. However, the difference in the identified SNPs between the two tools may be simply due to the small sample size of the study. Thus, further validation studies are warranted.

The strength of this study is that it is a GWAS that investigated genetic variants associated with heterogeneous cognitive function in a Korean PD population. However, there are several limitations of this study. First, the study currently lacks a replication study. To generalize the possible role of the *RYR2* variant for CI in PD, replication of the results in an independent sample is warranted. Second, the number of patients with MoCA scores was insufficient in our study (1). Some recent genomic studies on PD have revealed the significance of several genes for CI in PD, including α-synuclein (*SNCA*), beta-glucosidase (*GBA*), and *APOE*. However, these results were not replicated in our study (16). This difference between our results and those of previous studies could be due to genetic differences among the East Asian and Western PD populations or simply due to the aforementioned limitations of our study. Meta-analysis on future related studies and post-GWAS functional studies may increase our understanding in this field.

## Data Availability Statement

The original contributions presented in the study are publicly available. This data can be found here: https://www.ncbi.nlm.nih.gov/SNP/snp_viewBatch.cgi?sbid=1063218.

## Ethics Statement

The studies involving human participants were reviewed and approved by Institutional Review Board of Asan Medical Center. The patients/participants provided their written informed consent to participate in this study.

## Author Contributions

KWP executed the research project and statistical analysis and wrote the first draft of the manuscript. SYJ participated in the statistical analysis and data interpretation and reviewed the manuscript. MSK, SRJ, S-BK, and J-HL reviewed the statistical analysis and manuscript. HSR executed the research project and statistical analysis and reviewed the manuscript. J-HL and Y-MP executed the research project and reviewed the manuscript. SJC conceptualized and organized the research project and reviewed the statistical analysis and the manuscript. All authors contributed to the article and approved the submitted version.

## Conflict of Interest

The authors declare that the research was conducted in the absence of any commercial or financial relationships that could be construed as a potential conflict of interest.
