# Supplementary material for: Genomic Association Study for Cognitive Impairment in Parkinson's Disease
Source: Front Neurol. 2021 Feb 4;11:579268. doi: 10.3389/fneur.2020.579268 (PMC7890115; doi:10.3389/fneur.2020.579268)
Supplement: Supplementary file 1 [file Data_Sheet_1.docx]

**Supplementary Table 1. The top SNPs and genes with highest association from the analysis of 563,715 SNPs between patients with MMSE score<26 versus those with MMSE score≥26.**

| **CHR** | **BP** | **rs number** | **Gene** | **Region** | ***P*** | **OR** | **95% CI  (lower)** | **95% CI  (upper)** | **Ref** | **Alt** | **MAF** | **Physiologic role related to cognition** |
| --- | --- | --- | --- | --- | --- | --- | --- | --- | --- | --- | --- | --- |
| 1 | 237659147 | rs10495397 | *RYR2* | intron | 3.39E-06 | 3.21 | 1.96 | 5.25 | G | A | 0.04 | Neuroinflammation, calcium homeostasis^1-3^ |
| 2 | 118688596 | rs2288104 | *CCDC93* | intron | 1.70E-05 | 2.24 | 1.55 | 3.23 | T | C | 0.08 | Impaired retromer function^4^ |
| 5 | 160739709 | rs869648 | *GABRB2* | intron | 3.09E-05 | 1.94 | 1.42 | 2.66 | C | T | 0.11 | Alteration of GABAergic neurotransmission^5^ |
| 2 | 159749095 | rs146169520 | *TANC1* | upstream | 3.14E-05 | 4.27 | 2.16 | 8.46 | C | T | 0.02 | Regulate post-synaptic density by regulating  glutamatergic neurotransmission^6,7^ |

Abbreviations: CHR, chromosome; BP, base position; OR, odd's ratio; CI, confidence interval; Ref, reference allele; Alt, alternative allele; MAF, minor allele frequency.

**Supplementary Table 2. The top SNPs and genes with highest association from the analysis of 523,758 SNPs between patients with MoCAscore <24 versus those with MoCAscore ≥24.**

| **CHR** | **BP** | **rs number** | **Gene** | **Region** | ***P*** | **OR** | **95% CI  (lower)** | **95% CI  (upper)** | **Ref** | **Alt** | **MAF** | **Physiologic role related to cognition** |
| --- | --- | --- | --- | --- | --- | --- | --- | --- | --- | --- | --- | --- |
| 17 | 69676169 | rs2430514 | *CASC17* | downstream | 7.95E-06 | 2.03 | 1.49 | 2.77 | C | A | 0.44 | Unclear, but identified in GWAS on psychosis and impulsive behavior^8,9^ |
| 4 | 40358915 | rs10006948 | *CHRNA9* | downstream | 1.62E-05 | 2.54 | 1.66 | 3.88 | G | A | 0.16 | Neuronal cholinergic receptor^10^ |
| 17 | 32483237 | rs2228990 | *ASIC2* | synon | 2.48E-05 | 2.03 | 1.46 | 2.83 | G | A | 0.34 | Control acidosis-associated neuronal injury^11,12^ |
| 3 | 196613075 | rs34533379 | *SENP5* | synon,intron | 4.51E-05 | 2.56 | 1.63 | 4.02 | T | C | 0.14 | SUMOylation and regulation of DJ-1 protein^13,14^ |
| 16 | 77905658 | rs376490 | *VAT1L* | intron | 5.84E-05 | 2.31 | 1.54 | 3.48 | G | T | 0.17 | Identified in Alzheimer's disease GWAS^15^ |
| 8 | 4335204 | rs10101341 | *CSMD1* | intron | 6.88E-05 | 1.89 | 1.38 | 2.59 | A | C | 0.34 | Cell adhesion molecule involved in the development, connection, and plasticity of brain circuits^16,17^ |
| 3 | 184179373 | rs9870869 | *EIF2B5,EPHB3* | intron,downstream,upstream | 6.88E-05 | 1.88 | 1.38 | 2.57 | G | A | 0.36 | Regulate overall protein production,  cause of vanishing white matter disease (EIF2B5), axon-guidance pathway (EPHB3)^18,19^ |
| 1 | 37617809 | rs2884796 | *GRIK3* | upstream | 7.60E-05 | 2.30 | 1.52 | 3.48 | C | T | 0.18 | Glutamate ionotropic receptor^20^ |

Abbreviations: CHR, chromosome; BP, base position; OR, odds ratio; CI, confidence interval; Ref, reference allele; Alt, alternative allele; MAF, minor allele frequency.

**References**

1. Hopp SC, D'Angelo HM, Royer SE, Kaercher RM, Adzovic L, Wenk GL. Differential rescue of spatial memory deficits in aged rats by L-type voltage-dependent calcium channel and ryanodine receptor antagonism. *Neuroscience* 2014; **280**: 10-8.

2. Hopp SC, D'Angelo HM, Royer SE, et al. Calcium dysregulation via L-type voltage-dependent calcium channels and ryanodine receptors underlies memory deficits and synaptic dysfunction during chronic neuroinflammation. *J Neuroinflammation* 2015; **12**: 56.

3. Hopp SC, Royer SE, D'Angelo HM, Kaercher RM, Fisher DA, Wenk GL. Differential neuroprotective and anti-inflammatory effects of L-type voltage dependent calcium channel and ryanodine receptor antagonists in the substantia nigra and locus coeruleus. *J Neuroimmune Pharmacol* 2015; **10**(1): 35-44.

4. Cullen PJ, Steinberg F. To degrade or not to degrade: mechanisms and significance of endocytic recycling. *Nat Rev Mol Cell Biol* 2018.

5. Li Y, Sun H, Chen Z, Xu H, Bu G, Zheng H. Implications of GABAergic Neurotransmission in Alzheimer's Disease. *Front Aging Neurosci* 2016; **8**: 31.

6. Gasparini A, Tosatto SCE, Murgia A, Leonardi E. Dynamic scaffolds for neuronal signaling: in silico analysis of the TANC protein family. *Sci Rep* 2017; **7**(1): 6829.

7. Granot-Hershkovitz E, Raas-Rothschild A, Frumkin A, Granot D, Silverstein S, Abeliovich D. Complex chromosomal rearrangement in a girl with psychomotor-retardation and a de novo inversion: inv(2)(p15;q24.2). *Am J Med Genet A* 2011; **155A**(8): 1825-32.

8. Pappa I, St Pourcain B, Benke K, et al. A genome-wide approach to children's aggressive behavior: The EAGLE consortium. *Am J Med Genet B Neuropsychiatr Genet* 2016; **171**(5): 562-72.

9. Avramopoulos D, Pearce BD, McGrath J, et al. Infection and inflammation in schizophrenia and bipolar disorder: a genome wide study for interactions with genetic variation. *PLoS One* 2015; **10**(3): e0116696.

10. Richter K, Mathes V, Fronius M, et al. Phosphocholine - an agonist of metabotropic but not of ionotropic functions of alpha9-containing nicotinic acetylcholine receptors. *Sci Rep* 2016; **6**: 28660.

11. Jiang N, Wu J, Leng T, et al. Region specific contribution of ASIC2 to acidosis-and ischemia-induced neuronal injury. *J Cereb Blood Flow Metab* 2017; **37**(2): 528-40.

12. Lu Y, Ma X, Sabharwal R, et al. The ion channel ASIC2 is required for baroreceptor and autonomic control of the circulation. *Neuron* 2009; **64**(6): 885-97.

13. Shimizu Y, Lambert JP, Nicholson CK, et al. DJ-1 protects the heart against ischemia-reperfusion injury by regulating mitochondrial fission. *Journal of molecular and cellular cardiology* 2016; **97**: 56-66.

14. Guerra de Souza AC, Prediger RD, Cimarosti H. SUMO-regulated mitochondrial function in Parkinson's disease. *Journal of neurochemistry* 2016; **137**(5): 673-86.

15. Sherva R, Tripodis Y, Bennett DA, et al. Genome-wide association study of the rate of cognitive decline in Alzheimer's disease. *Alzheimers Dement* 2014; **10**(1): 45-52.

16. Athanasiu L, Giddaluru S, Fernandes C, et al. A genetic association study of CSMD1 and CSMD2 with cognitive function. *Brain, behavior, and immunity* 2017; **61**: 209-16.

17. Koiliari E, Roussos P, Pasparakis E, et al. The CSMD1 genome-wide associated schizophrenia risk variant rs10503253 affects general cognitive ability and executive function in healthy males. *Schizophrenia research* 2014; **154**(1-3): 42-7.

18. Lesnick TG, Papapetropoulos S, Mash DC, et al. A genomic pathway approach to a complex disease: axon guidance and Parkinson disease. *PLoS Genet* 2007; **3**(6): e98.

19. Lin L, Lesnick TG, Maraganore DM, Isacson O. Axon guidance and synaptic maintenance: preclinical markers for neurodegenerative disease and therapeutics. *Trends Neurosci* 2009; **32**(3): 142-9.

20. Takenouchi T, Hashida N, Torii C, Kosaki R, Takahashi T, Kosaki K. 1p34.3 deletion involving GRIK3: Further clinical implication of GRIK family glutamate receptors in the pathogenesis of developmental delay. *Am J Med Genet A* 2014; **164a**(2): 456-60.
